# Supplementary figures and images for: Optimization of cytotoxic activity of Nocardia sp culture broths using a design of experiments
Source: PLoS One. 2020 Jan 14;15(1):e0227816. doi: 10.1371/journal.pone.0227816 (PMC6959983; doi:10.1371/journal.pone.0227816)

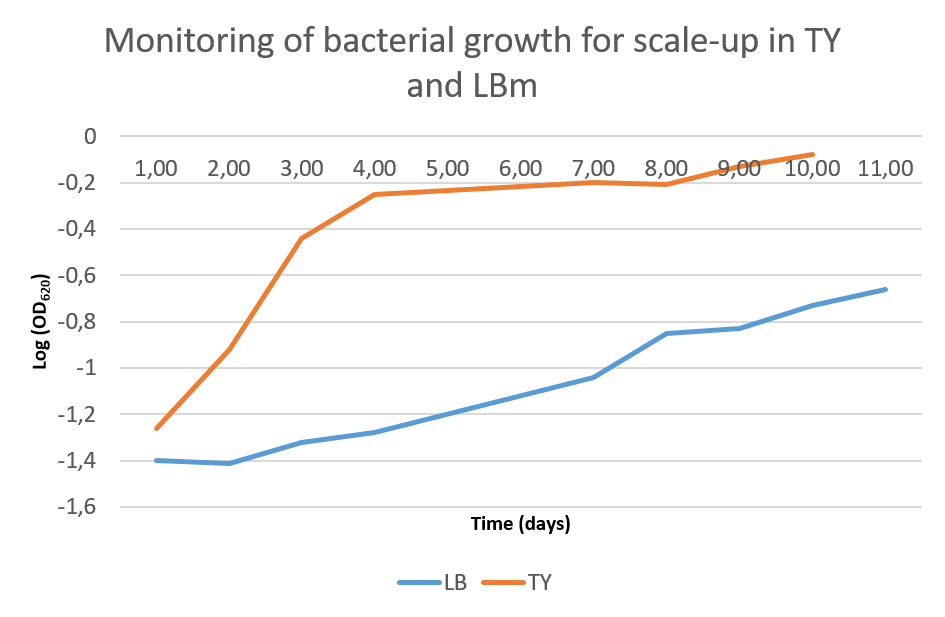

Supplement: S1 Fig — (TIF) [file pone.0227816.s001.tif]

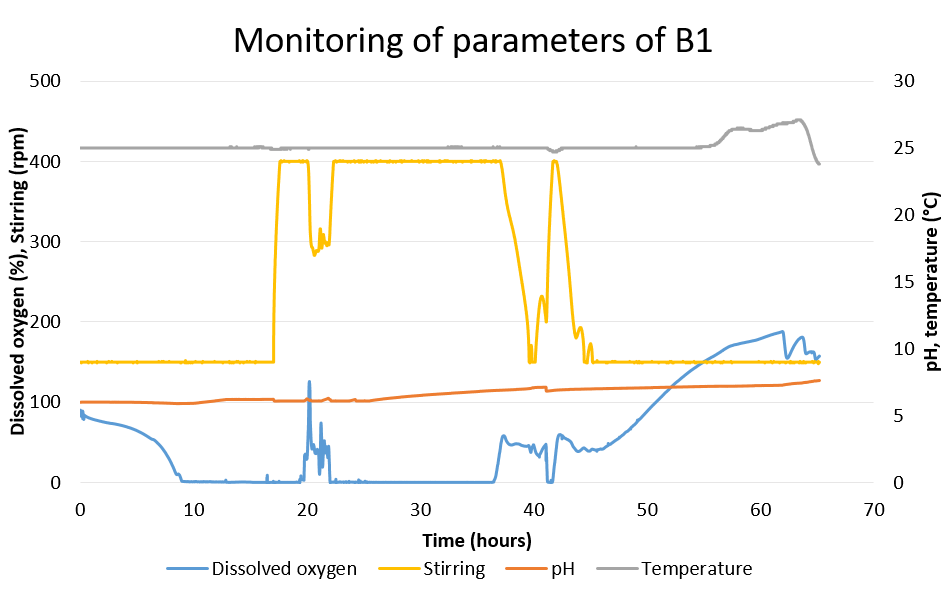

Supplement: S2 Fig — (TIF) [file pone.0227816.s002.tif]

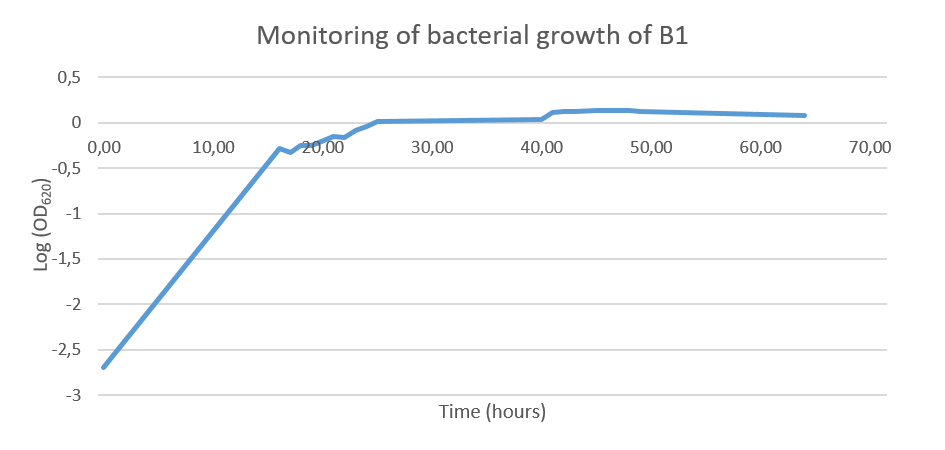

Supplement: S3 Fig — (TIF) [file pone.0227816.s003.tif]

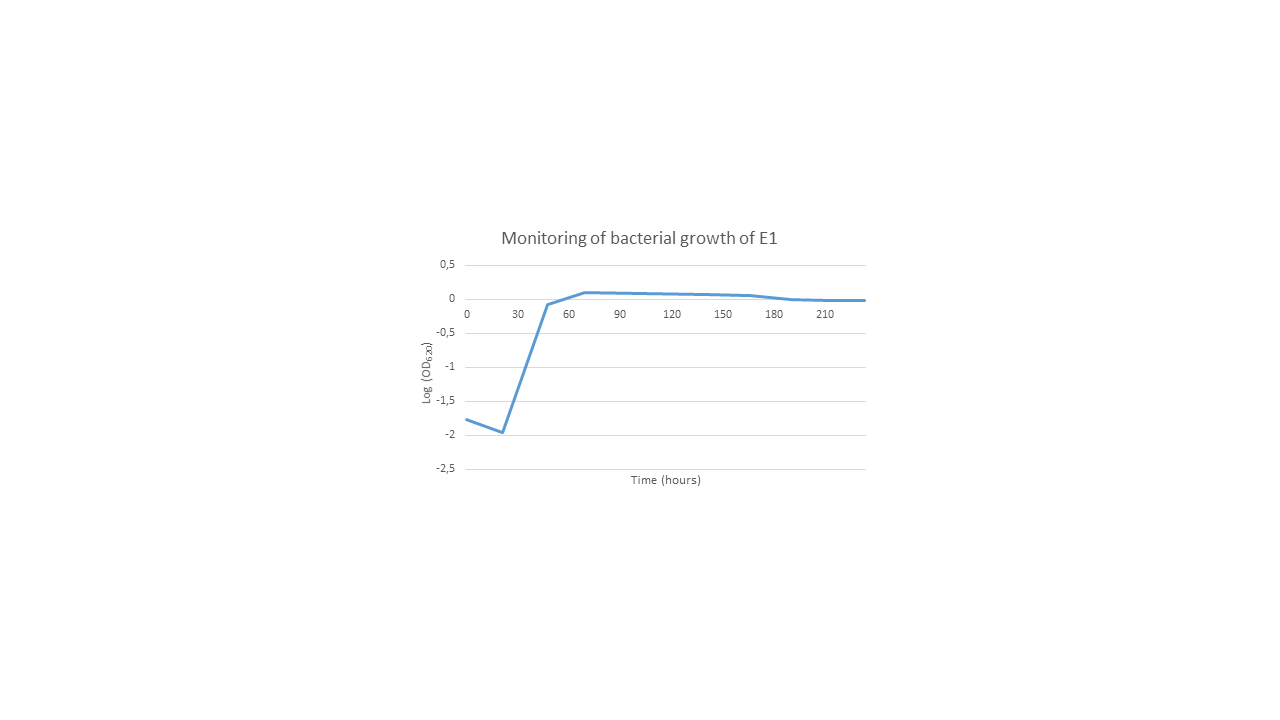

Supplement: S4 Fig — (TIF) [file pone.0227816.s004.tif]
