# Supplementary material for: Optimization of cytotoxic activity of Nocardia sp culture broths using a design of experiments
Source: PLoS One. 2020 Jan 14;15(1):e0227816. doi: 10.1371/journal.pone.0227816 (PMC6959983; doi:10.1371/journal.pone.0227816)
Supplement: Attachment — Submitted filename: PONE-D-19-18890_reviewer#1.pdf [file pone.0227816.s005.pdf]

PLOS ONE

Optimization of cytotoxic activity of Nocardia sp culture broths using a design of experiments

--Manuscript Draft--

**Manuscript Number:**	PONE-D-19-18890
**Article Type:**	Research Article
**Full Title:**	Optimization of cytotoxic activity of Nocardia sp culture broths using a design of experiments
**Short Title:**	Design of experiments for optimization of Nocardia culture
**Corresponding Author:**	sophie Tomasi<br>Institut des Sciences Chimiques de Rennes<br>Rennes cedex, FRANCE
**Keywords:**	Culture optimization; cytotoxicity; experimental design; lichen-associated bacteria; OSMAC.
**Abstract:**	Background<br><br>Lichen-associated bacteria represent an underexplored potential source for the discovery of novel compounds. To induce the production of cytotoxic compounds we applied an OSMAC (One Strain Many Compounds) approach on Nocardia sp which was selected among several strains isolated from Lathagrium auriforme (ex Collema auriforme).<br><br>Results<br><br>Bacteria were cultured under different conditions and two different extracts, a supernatant and a XAD-7HP resin extracts were obtained.<br>Preliminary assays, performed with extracts from four different culture media (modified LB, TY, MB, ISP2), led to the selection of an extract from TY culture with interesting cytotoxicity against two cell lines (HaCaT: IC50 = 8.0 ± 1.5 µg/mL; B16: IC50 = 4.6 ± 1.8 µg/mL) but with a low yield (16.1 mg/L).<br>Using Taguchi L9 orthogonal array design in TY culture medium, the increase in cytotoxic activity of extracts obtained from two different vessels (bioreactor or Erlenmeyer flasks) was investigated. Varying culture parameters such as, temperature, pH and inoculum ratio (%) were used. For experiments conducted in a bioreactor, stirring speed was included as an additional parameter.<br><br>Conclusions<br><br>Significant differences in the cytotoxic activities of different extracts on B16 cell lines, highlighted the influence of temperature on the production of cytotoxic compound(s) in a bioreactor. A culture in Erlenmeyer flasks was also performed and afforded an increase of the production of the active compounds. The best conditions for the highest cytotoxicity (IC50 on B16: 6 ± 0.5 µg/mL) and the highest yield (202.0 mg/L) were identified as: pH 6, temperature 37°C and 5% inoculum.
**Order of Authors:**	sophie Tomasi
	Gwendoline Van Soen
	Isabelle Rouaud
	Eric Hitti
	sophie Tomasi
**Additional Information:**	
**Question**	**Response**
**Financial Disclosure**	The funders had no role in study design, data collection and analysis, decision to

Enter a financial disclosure statement that describes the sources of funding for the work included in this submission. Review the submission guidelines for detailed requirements. View published research articles from *PLOS ONE* for specific examples.<br><br>This statement is required for submission and **will appear in the published article** if the submission is accepted. Please make sure it is accurate.<br><br>**Unfunded studies**<br>Enter: *The author(s) received no specific funding for this work.*<br><br>**Funded studies**<br>Enter a statement with the following details:<br>• Initials of the authors who received each award<br>• Grant numbers awarded to each author<br>• The full name of each funder<br>• URL of each funder website<br>• Did the sponsors or funders play any role in the study design, data collection and analysis, decision to publish, or preparation of the manuscript?<br>• **NO** - Include this sentence at the end of your statement: *The funders had no role in study design, data collection and analysis, decision to publish, or preparation of the manuscript.*<br>• **YES** - Specify the role(s) played.<br><br>* typeset	publish, or preparation of the manuscript.
**Competing Interests**<br><br>Use the instructions below to enter a competing interest statement for this submission. On behalf of all authors, disclose any competing interests that could be perceived to bias this work—acknowledging all financial support and any other relevant financial or non-financial competing interests.<br><br>This statement **will appear in the published article** if the submission is accepted. Please make sure it is accurate. View published research articles	The authors have declared that no competing interests exist.

from *PLOS ONE* for specific examples.<br><br>**NO authors have competing interests**<br><br>Enter: *The authors have declared that no competing interests exist.*<br><br>**Authors with competing interests**<br><br>Enter competing interest details beginning with this statement:<br><br>*I have read the journal's policy and the authors of this manuscript have the following competing interests: [insert competing interests here]*<br><br>* typeset	
**Ethics Statement**<br><br>Enter an ethics statement for this submission. This statement is required if the study involved:<br><br>• Human participants<br>• Human specimens or tissue<br>• Vertebrate animals or cephalopods<br>• Vertebrate embryos or tissues<br>• Field research<br><br>Write "N/A" if the submission does not require an ethics statement.<br><br>General guidance is provided below. Consult the submission guidelines for detailed instructions. **Make sure that all information entered here is included in the Methods section of the manuscript.**	N/A

**Format for specific study types**<br><br>**Human Subject Research (involving human participants and/or tissue)**<br>• Give the name of the institutional review board or ethics committee that approved the study<br>• Include the approval number and/or a statement indicating approval of this research<br>• Indicate the form of consent obtained (written/oral) or the reason that consent was not obtained (e.g. the data were analyzed anonymously)<br><br>**Animal Research (involving vertebrate animals, embryos or tissues)**<br>• Provide the name of the Institutional Animal Care and Use Committee (IACUC) or other relevant ethics board that reviewed the study protocol, and indicate whether they approved this research or granted a formal waiver of ethical approval<br>• Include an approval number if one was obtained<br>• If the study involved *non-human primates*, add *additional details* about animal welfare and steps taken to ameliorate suffering<br>• If anesthesia, euthanasia, or any kind of animal sacrifice is part of the study, include briefly which substances and/or methods were applied<br><br>**Field Research**<br><br>Include the following details if this study involves the collection of plant, animal, or other materials from a natural setting:<br>• Field permit number<br>• Name of the institution or relevant body that granted permission	
**Data Availability**<br><br>Authors are required to make all data underlying the findings described fully available, without restriction, and from the time of publication. PLOS allows rare exceptions to address legal and ethical concerns. See the PLOS Data Policy and FAQ for detailed information.	Yes - all data are fully available without restriction

A Data Availability Statement describing where the data can be found is required at submission. Your answers to this question constitute the Data Availability Statement and **will be published in the article**, if accepted.<br><br>**Important:** Stating 'data available on request from the author' is not sufficient. If your data are only available upon request, select 'No' for the first question and explain your exceptional situation in the text box.<br><br>Do the authors confirm that all data underlying the findings described in their manuscript are fully available without restriction?	
**Describe where the data may be found in full sentences. If you are copying our sample text, replace any instances of XXX with the appropriate details.**<br><br>• If the data are **held or will be held in a public repository**, include URLs, accession numbers or DOIs. If this information will only be available after acceptance, indicate this by ticking the box below. For example: *All XXX files are available from the XXX database (accession number(s) XXX, XXX.*).<br>• If the data are all contained **within the manuscript and/or Supporting Information files**, enter the following: *All relevant data are within the manuscript and its Supporting Information files.*<br>• If neither of these applies but you are able to provide **details of access elsewhere**, with or without limitations, please do so. For example:<br><br>*Data cannot be shared publicly because of [XXX]. Data are available from the XXX Institutional Data Access / Ethics Committee (contact via XXX) for researchers who meet the criteria for access to confidential data.*<br><br>*The data underlying the results presented in the study are available from (include the name of the third party*	All relevant data are within the manuscript and its Supporting Information files.

and contact information or URL).<br>• This text is appropriate if the data are owned by a third party and authors do not have permission to share the data.<br><br>* typeset	
Additional data availability information:	Tick here if the URLs/accession numbers/DOIs will be available only after acceptance of the manuscript for publication so that we can ensure their inclusion before publication.

Click here to download Manuscript Publi-PlosOne-ST-AN.docx

Research Article

Optimization of cytotoxic activity of *Nocardia* sp culture broths using a design of experiments

Alba Noël[1#], Gwendoline Van Soen[1], Isabelle Rouaud[1], Eric Hitti[2], Sophie Tomasi[1*].

[1] Univ Rennes, CNRS, ISCR – UMR 6226, Rennes, France

[2] LTSI, UMR_S 1099, UFR Sciences Pharmaceutiques et Biologiques, Rennes, France

[#] Current address: I2BC, CNRS – UMR 9198, Orsay, France

*** Corresponding author:**

E-mail: sophie.tomasi@univ-rennes1.fr (ST)

Abstract

Background: Lichen-associated bacteria represent an underexplored potential source for the discovery of novel compounds. To induce the production of cytotoxic compounds we applied an OSMAC (One Strain Many Compounds) approach on *Nocardia* sp which was selected among several strains isolated from *Lathagrium auriforme* (ex *Collema auriforme*).

Results: Bacteria were cultured under different conditions and two different extracts, a supernatant and a XAD-7HP resin extracts were obtained.

Preliminary assays, performed with extracts from four different culture media (modified LB, TY, MB, ISP2), led to the selection of an extract from TY culture with interesting cytotoxicity against two cell lines (HaCaT: IC_{50} = 8.0 ± 1.5 µg/mL; B16: IC_{50} = 4.6 ± 1.8 µg/mL) but with a low yield (16.1 mg/L).

Using Taguchi L9 orthogonal array design in TY culture medium, the increase in cytotoxic activity of extracts obtained from two different vessels (bioreactor or Erlenmeyer flasks) was investigated. Varying culture parameters such as, temperature, pH and inoculum ratio (%) were used. For experiments conducted in a bioreactor, stirring speed was included as an additional parameter.

Conclusions: Significant differences in the cytotoxic activities of different extracts on B16 cell lines, highlighted the influence of temperature on the production of cytotoxic compound(s) in a bioreactor. A culture in Erlenmeyer flasks was also performed and afforded an increase of the production of the active compounds. The best conditions for the highest cytotoxicity (IC_{50} on B16: 6 ± 0.5 µg/mL) and the highest yield (202.0 mg/L) were identified as: pH 6, temperature 37°C and 5% inoculum.

Introduction

Some antibiotics such as actinomycin, streptothricin and streptomycin have been discovered from Actinobacteria since the forties [1]. These organisms also produce a wide variety of secondary metabolites with interesting biological activities such as insecticidal (e.g. avermectins from *S. avermitilis* [2]), antifungal (e.g. amphotericin B from *S. nodosus* [3]), antiviral (e.g. hygromycin from *S. hygroscopicus* [4]), and cytotoxic activities (e.g. bleomycin from *Streptomyces verticillus* [5] or asterobactin from *Nocardia asteroides* [6]). These bacteria are also well described for their interesting biotechnological applications [7]. Moreover they are widespread in environment making them accessible sources of novel bioactive metabolites, as recently reported with Streptomyces and Nocardia strains isolated from underground lake and moonmilk [8].

Microbial population associated with lichens have been recently characterized in the lichen symbiosis classically defined like a dual symbiosis between a green alga (or a cyanobacterium) and a fungus [9]. Indeed, a plethora of bacteria have been identified from lichens using culture-independent (reviewed by Suzuki et al [10]) or culture-dependent approaches [11,12] and their bacterial community seems to be specific to the lichen species [13]. This long-lasting ecological niche is a novel reservoir for interesting bacterial strains. The three main bacterial phyla associated with lichens are Proteobacteria, Firmicutes and Actinobacteria [12–14]. The chemical production of several strains has already been described [10,15] highlighting their ability to produce active compounds.

In this study, an Actinobacterium isolated from a terrestrial lichen species *Lathagrium auriforme* [12] (ex *Collema auriforme*), close to *Nocardia* sp. (100% 16S rRNA sequence similarity with *Nocardia soli*, *N. cummidelens* and *N. salmonicida* and 98.59% with *N. ignorata*), was used. To our knowledge only our previous study has described the chemical production of this *Nocardia* strain [16]. A preliminary assay showed that the culture broth extract of this bacterium possessed an interesting cytotoxic activity against B16 (murine

melanoma) cell lines (IC_{50} = 23 ± 3 µg/mL). The aim of this study was to estimate the impact of different culture parameters on this cytotoxicity. We have chosen herein to evaluate various parameters: pH, temperature, stirring speed and the inoculum ratio (%) (v/v). The evaluation of all these parameters requires 81 experiments, which is very time consuming. A Taguchi L9 orthogonal array design, which is a robust statistical DOE (Design of Experiments) method, was employed to perform 9 experiments instead of 81 [17]. At the end of the stationary growth phase the cultures were stopped and the yield and the cytotoxic activity of the extracts were measured. ANOVA (Analysis Of Variance) test was used to highlight which parameters exhibited a significant influence on the cytotoxicity of the bacterial extracts. Finally, the chemical analyses of the most active extracts were also carried out using HPLC (High Performance Liquid Chromatography) in order to highlight the common metabolites.

Materials and methods

Microorganism

The strain used for this study was isolated from a terrestrial lichen *Lathagrium auriforme* (ex *Collema auriforme*) collected in Kesselfallklamm in Austria (47°12'21.26" N, 15°23'57.27" E) in November 2012 by Parrot et al [12]. Its 16S rRNA gene was then sequenced using Sanger sequencing (874 pb) and the close phylogenetic neighbors of our strain were identified as *Nocardia ignorata* DQ659907 at 98.59% sequence identity by comparison of these data with sequences in the Eztaxon server type strain database [18]. A second sequencing of 993 pb following the same protocol as described by Parrot et al [12] allowed the identification of three closer phylogenetic neighbors *Nocardia soli*, *N. cummidelens* and *N. salmonicida* at 100% sequence identity using the Eztaxon server. The

strain was stored after growth in ISP2 medium [19] with 50% v/v glycerol or 5% v/v DMSO (Dimethylsulfoxyde) at −80°C and referenced as DP94 [12]. This strain was deposited at the Institute of Plant Sciences, University of Graz

Preliminary assays

Small-scale cultures

Four media cited below were used for preliminary assays:

- TY (Tryptone Yeast medium): 10 g/L yeast extract (Sigma-Aldrich, St Louis, MO, USA), 16 g/L tryptone (Sigma-Aldrich, St Louis, MO, USA), and 5 g/L NaCl (Sigma-Aldrich, St Louis, MO, USA),
- Modified LB (Lysogeny Broth) (LBm): 5 g/L peptone (Sigma-Aldrich, St Louis, MO, USA), 5 g/L NaCl (Sigma-Aldrich, St Louis, MO, USA) and 3 g/L yeast extract (Sigma-Aldrich, St Louis, MO, USA)
- ISP2 (International Streptomyces Project medium 2): 4 g/L yeast extract (Sigma-Aldrich, St Louis, MO, USA), 10 g/L malt extract (Sigma-Aldrich, St Louis, MO, USA) and 4 g/L glucose
- MB (Marine Broth): 37.4 g/L of commercial marine broth (Difco ® 2216, ThermoFisher, Waltham, MA, USA)

30 mL of each medium were inoculated in duplicate with the bacterial strain and incubated at 25°C and at 110 rpm in an incubator New Brunswick® Innova 42 (New Brunswick®, Edison, NJ, USA). After 8, 11 or 14 days of incubation the culture broth was centrifuged at 3000 rpm (Thermo scientific Sorvall ST40R, ThermoFisher scientific, Waltham, MA, USA), at 4°C for 15 min. The supernatant was extracted with 2 x 30 mL of ethyl acetate and the organic layer was dried on anhydrous sodium sulfate and the solvent was evaporated *in vacuo* leading to a raw extract.

Scale-up process

5.4 L of TY medium and 7.2 L of LBm medium were divided in 500 mL Erlenmeyer flasks and inoculated with 1% of preculture of the bacterial strain. The cultures were incubated at 25 °C, 110 rpm in an incubator New Brunswick® Innova 42 (New Brunswick®, Edison, NJ, USA). After 11 days of incubation the culture broth was extracted as described below. The monitoring of the bacterial growth of these scale-up cultures is reported in S1 Fig.

Culture extraction

After fermentation the broth was collected and centrifuged at 3000 rpm (Thermo scientific Sorvall ST40R, ThermoFisher scientific, Waltham, MA, USA), at 4°C for 15 min. The supernatant was collected and 40 g of a resin Amberlite® XAD 7HP (Sigma-Aldrich, St Louis, MO, USA) were added per liter. After 4h of stirring at 150 rpm the resin was filtered and desorbed three times successively with 400 mL of a mixture of MeOH/acetone (1/1, v/v) per 40 g of resin. The solvents were evaporated *in vacuo* and the residue was dissolved in 100 mL of water and extracted with 2 x 100 mL of EtOAc. The organic layer was then dried with anhydrous sodium sulfate, filtered and evaporated *in vacuo* leading to the resin extract (RE). The supernatant previously treated with the resin was extracted two times with EtOAc (1/2, v/v) and the organic layer was dehydrated with anhydrous sodium sulfate, filtered and dried *in vacuo* leading to the supernatant extract (SE). An extraction yield was defined as follows:

$$\text{Yield} = \frac{\text{masse of the extract (mg)}}{\text{volume of culture medium (L)}}$$

Culture in bioreactor

Fermentation was performed in 2.5 L of TY medium in a bioreactor BioFlo/Celligen® 115 (New Brunswick®, Edison, NJ, USA). The oxygenation rate of the medium was regulated at 0.4 vvm (volume of gas per volume of liquid per minute) and the percentage of dissolved oxygen was maintained up to 40% by using the cascade mode with the stirring. Temperature, pH, stirring and inoculum ratio (%) (v/v) were selected according to the design of experiments (Tables 1 and 2). The cultures were stopped after 5 days of fermentation. An example of monitoring of culture parameters and of bacterial growth has been reported in S2 and S3 Figs.

Culture in Erlenmeyer flasks

Fermentation of the strain was performed in 300 mL of TY medium in 500 mL Erlenmeyer flask. The pH of the medium was adjusted with a solution of NaOH 5 M or H_2SO_4 0.5 N. Flasks were then incubated in an incubator New Brunswick® Innova 42 (New Brunswick®, Edison, NJ, USA) with a stirring fixed at 150 rpm and a temperature fixed at a value according to the design of experiments (See Tables 8, 9, 10 and 11). The cultures were stopped after 11 days of fermentation. The monitoring of bacterial growth was performed by measurement of OD (Optical Density) as shown in SI4.

Taguchi experimental design

The Taguchi method was selected to establish the experimental design and led to 9 experiments either in bioreactor (Tables 1 and 2) or in Erlenmeyer flask (Tables 3 and 4). For each experiment, the responses evaluated were the yield and the cytotoxicity of extracts against B16 and HaCaT cell lines. For the fermentation in bioreactor we selected 4

independent parameters and attributed 3 levels for each. The four parameters selected were temperature (25°C, 30°C and 37°C), pH (6, 7 and 8), percentage of inoculum (v/v) (1%, 2% and 5%) and stirring speed (150, 200 and 250 rpm) (Table 1).

Table 1. Assignment of factors and level setting of the orthogonal array design L9 (3^3) for culture in bioreactor.

Factors	Level 1	Level 2	Level 3
**[A] Temperature (°C)**	25	30	37
**[B] pH**	6	7	8
**[C] inoculum %**	1	2	5
**[D] stirring speed (rpm)**	150	200	250

Table 2. Assignment of the experimental conditions in the orthogonal design L9 (3^4) for cultures in bioreactor.

Exp n°	Factor[a]			
	[A]	[B]	[C]	[D]
**B1**	1	1	1	1
**B2**	2	1	2	2
**B3**	3	1	3	3
**B4**	1	2	2	3
**B5**	2	2	3	1
**B6**	3	2	1	2
**B7**	1	3	3	2
**B8**	2	3	1	3
**B9**	3	3	2	1

[a][A]: Temperature, [B]: pH, [C]: inoculum ratio (%), [D]: stirring

For the fermentation in Erlenmeyer flasks the stirring was fixed at 150 rpm and only 3 parameters were selected with 3 levels for each (Tables 3 and 4).

Table 3. Assignment of factors and level setting of the orthogonal array design L9 (3^3) for culture in Erlenmeyer flasks.

Factors	Level 1	Level 2	Level 3
**[A] Temperature (°C)**	25	30	37
**[B] pH**	6	7	8
**[C] inoculum %**	1	2	5

Table 4. Experimental conditions in the orthogonal design L9 (3^3) for culture in Erlenmeyer flasks.

**Exp n°**	**Factor[a]**		
	**[A]**	**[B]**	**[C]**
**E1**	1	1	1
**E2**	1	2	2
**E3**	1	3	3
**E4**	2	1	2
**E5**	2	2	3
**E6**	2	3	1
**E7**	3	1	3
**E8**	3	2	1
**E9**	3	3	2

[a] [A]: Temperature, [B]: pH, [C]: inoculum ratio (%)

Cytotoxic assays

The cytotoxic activity of the resin extracts and the supernatant extracts was evaluated for each experiment of the experimental design with a standard tetrazolium assay (MTT) [20]. Two cell lines (B16: murine melanoma, HaCaT: human keratinocytes) were cultivated in RPMI (Roswell Park Memorial Institute m[illegible]um) 1640 medium (Thermo Fisher Scientific, USA) supplemented with 5% fetal calf serum (FCS) and 1% antibiotics (penicillin 10 000 UI/ml and streptomycin 10 000 µg/ml, Eurobio®, Les Ulis, France). Cells were then seeds

in 96-wells plate (B16: 6000 cells/well and HaCaT 10,000 cells/well) at day 0 and incubated for 24h at 37°C in an atmosphere of 5% CO_2. Extracts were then added at different concentrations (1, 10, 50, 100, and 200 μg/mL) for all the experiments. For experimental design in bioreactor and Erlenmeyer flasks the extracts were tested a second time at 5, 50, 100, 250 and 500 μg/mL to expand the range of IC_{50} values. After 24h of incubation (37°C, 5% CO_2) the cell viability was measured at 540 nm using a MTT (3-[4,5-dimethylthiazol-2-yl]-2,5-diphenyltetrazolium bromide) assay [20]. Each experiment was repeated three times.

Chemical analyses

Chemical analyses of the most active extracts were carried out on a HPLC system—Diode Array Detector (LC-DAD) (Shimadzu, Marne La Vallée, France). A Prevail C18 column (5 µm, 250 × 4.6 mm, GRACE, Columbia, MD, USA) was used and a gradient system was applied: A (0.1% formic acid in water) and B (0.1% formic acid in acetonitrile). The following gradient was applied at a flow rate of 0.8 mL/min in the HPLC system: initial: 100% (A); from 0 to 5 min: 100% (A); from 5 to 35 min: 100% (A)/0% (B) to 0% (A)/100% (B); from 35 to 45 min: 100% B; from 45 to 50 min: 100% (A)/0% (B) to 0% (A)/100% (B); from 50 to 55 min: 100% (A). Samples were prepared by dissolving extracts in MeOH at 1 mg/mL and 20 µL were injected after filtration (45 µm). The LabSolutions software (Shimadzu, Marne La Vallée, France) was used for data analyses.

Results

Preliminary assays

The *Nocardia* sp. strain used in this study achieved the stationary growth phase after 7 days of growth. We have cultivated this strain using 30 mL of four different media in 50 mL tubes, with a 7 days of exponential growth phase followed by 1, 4 or 7 days of stationary phase (respectively 8, 11 and 14 days in total). The best cytotoxic activity against B16 cells was achieved with the culture in LBm medium for 11 days (IC_{50} = 8 ± 2 µg/mL) and with the ISP2 medium after 14 days of culture (IC_{50} = 7.5 ± 0.5 µg/mL). Similar activities have been shown on HaCaT cells. The highest amount of extract was obtained when the strain was grown in TY medium (Table 5). A scale-up assay with 300 mL of media in 500 mL Erlenmeyer flasks was then performed using LBm and TY for 11 days of culture (4 days of stationary phase). In order to have sufficient amount of LBm extracts and based on the results obtained with the 30 mL process, we have inoculated 24 Erlenmeyer flasks with LBm medium (total volume 7.2 L) and 18 Erlenmeyer flasks with TY medium (total volume 5.4 L). The culture extract from TY medium gave the best activity against B16 cell line (IC_{50} = 4.6 ± 1.8 µg/mL) even though the amount obtained was low (yield = 16.1 mg/L) but higher than for the culture in LBm (Table 6). These culture conditions (TY medium and 4 days of stationary phase) were applied to the Taguchi design experiments.

Table 5. Weight and cytotoxicity of extracts in preliminary assays.

Days of culture	Medium[a]	Raw extract amount (mg)	IC_{50} (μg/mL)	
			B16	HaCaT
8	ISP2	2.3 ± 0.0	45 ± 5	59 ± 5
	TY	5.2 ± 0.1	41 ± 5	72 ± 7
	MB	2.2 ± 0.3	105 ± 20	115 ± 15
	LBm	2.5 ± 0.4	25 ± 4	95 ± 30
11	ISP2	4.0 ± 0.7	31 ± 10	64 ± 28
	TY	**5.4 ± 0.9**	**25 ± 4**	**47 ± 13**
	MB	2.1 ± 0.7	57 ± 18	120 ± 15
	LBm	**2.5 ± 0.1**	**8 ± 2**	**31 ± 12**
14	ISP2	**3.6 ± 0.2**	**7.5 ± 0.5**	**41 ± 5**
	TY	6.4 ± 0.3	35 ± 8	86 ± 42
	MB	3.8 ± 0.2	85 ± 10	79 ± 9
	LBm	4.2 ± 0.2	14 ± 6	70 ± 4

[a]ISP2 = International Streptomyces Project 2, TY = Tryptone Yeast, MB = Marine Broth, LBm = modified Lysogeny Broth

Table 6. Extraction yield and cytotoxicity of extracts from scale up in Erlenmeyer flasks.

Medium	Extract	Yield (mg/L)	IC_{50} (μg/mL)	
			B16	HaCaT
TY	Resin extract	101.4	>200	>200
	Supernatant extract	16.1	**4.6 ± 1.8**	**8 ± 1.5**
LBm	Resin extract	60.3	>200	>200
	Supernatant extract	3.0	52 ± 20	73 ± 20

Experimental design in bioreactor

Results

An experimental design was applied in order to find the best culture conditions to improve the yield and the cytotoxic activity of extracts. Varying temperature [A] (25, 30 and 37 °C), pH [B] (6, 7 and 8), percentage of inoculum ratio (% v/v) [C] (1, 2 and 5%) and stirring speed [D] (150, 200 and 250 rpm), 9 experiments were carried out. In all experiments, the yield of the resin extracts (RE) and the supernatant extracts (SE) were determined and their cytotoxicity was measured against B16 and HaCaT cell lines (Table 7). When similar activities have been exhibited against the two cell lines, we have focused on activities against B16.

Table 7. Extraction yield and cytotoxicity of the extracts from the experimental design using bioreactor.

**Exp n°**	**RE**			**SE**		
	**Yield (mg/L)**	**IC_{50} (µg/mL)**		**Yield (mg/L)**	**IC_{50} (µg/mL)**	
		**B16**	**HaCaT**		**B16**	**HaCaT**
**B1**	146.1	157 ± 77	92 ± 29	46.3	>500	460 ± 140
**B2**	160.5	205 ± 100	185 ± 65	1859.3	>500	>500
**B3**	**216.6**	**53 ± 16**	**152 ± 28**	**478.6**	**41 ± 8**	**20 ± 8**
**B4**	158.0	92 ± 19	70 ± 8	152.3	250	320
**B5**	177.8	345 ± 95	120 ± 40	506.9	100 ± 30	150
**B6**	223.5	45 ± 15	80 ± 20	36.1	270 ± 35	225 ± 45
**B7**	126.8	270 ± 30	185 ± 15	66.5	430 ± 90	180 ± 20
**B8**	703.0	340 ± 25	397 ± 27	18.4	67 ± 4	38 ± 19
**B9**	318.0	90 ± 50	110 ± 25	43.9	350	270

The mean IC_{50} values of RE on B16 cells, were classified into three groups: a first group with IC_{50} > 300 µg/mL (exp. **B5** and **B8**); a second group with 100 < IC_{50} < 300 µg/mL (exp. **B1**, **B2** and **B7**) and the last group with IC_{50} < 100 µg/mL (exp. **B3**, **B4**, **B6** and **B9**) (Table 7). In a similar manner, the results were classified into three groups for SE extracts based on their activity on B16: the first group with IC_{50} > 400 µg/mL (exp. **B1**, **B2** and **B7**); a second group with 100 < IC_{50} < 400 µg/mL (exp. **B4**, **B6** and **B9**) and the last group with IC_{50} < 100 µg/mL (exp. **B3**, **B5** and **B8**) (Table 7).

Most of RE extracts exhibited cytotoxic activities whereas only a few of SE extracts were active. Main-effect plots (Fig 1) indicated that the IC_{50} values decreased with the maximum level of factor B at 37°C. It was also noticed that the IC_{50} values increased in culture extracts at 30°C, highlighting the importance of culture temperature on the cytotoxicity of the extracts.

Finally, the experiment with the highest activity was the experiment **B3** (RE, IC_{50} = 53 ± 16 µg/mL and SE IC_{50} = 41 ± 8 µg/mL on B16) with the following culture conditions: pH 6, temperature 37°C, an inoculum of 5% and a stirring of 250 rpm.

Surprisingly, the experiment **B2** (culture conditions: pH 6, temperature of 30°C, inoculum of 2% and stirring speed of 200 rpm) gave the highest extraction yield.

Based on the cytotoxic activity against B16 and the extraction yield, ANOVA test was used to highlight which parameter(s) could significantly influence the production of active compound(s).

Fig 1. Main-effect plots for optimization of culture conditions in bioreactor.

Statistical analysis

The F-ratio and the p-value determined from the ANOVA analysis on the cytotoxicity of the resin extracts showed that the temperature significantly influenced the activity ($p < 0.05$)

(Table 8 and Fig 1). Conversely, none of the parameters selected for this analysis showed a significant effect on the extraction yield as well as for the cytotoxicity of the supernatant extracts (Table 9).

Table 8. Summary of analysis of variance in the ANOVA test for cytotoxicity on B16 of RE from bioreactor.

Factors[a]	Sum of squares	f	Average of square	F-ratio	F0.95(2,2)	p
**A**	14804.2	2	7402.1	7.46	19	0.118
**B**	82222.9	2	41111.4	41.44	19	0.024
**C**	1984.2	2	992.1	1.00	19	0.500
**D**	13206.9	2	6603.4	6.66	19	0.131
**Error**	1984.2	2	992.1			
**Total**	112218.2					

[a] [A]: Temperature, [B]: pH, [C]: inoculum ratio (%), [D]: stirring

Table 9. Summary of p-value from the ANOVA test on all extracts from bioreactor.

Factors[a]	p			
	RE		SE	
	Effect on IC_{50} on B16	Effect on extraction yield	Effect on IC_{50} on B16	Effect on extraction yield
**A**	0.118	0.408	0.500	0.303
**B**	0.024	0.475	0.333	0.312
**C**	0.500	0.490	0.192	0.500
**D**	0.131	0.500	0.388	0.385

[a] [A]: Temperature, [B]: pH, [C]: inoculum ratio (%), [D]: stirring

Validation of the optimal conditions

The experiment **B3** with the following parameters, pH 6, temperature 37°C, inoculum 5% and stirring 250 rpm, was repeated using bioreactor to confirm the previous results on

HaCaT and B16. The repetition of this experiment showed close values of IC_{50} for SE on B16 and HaCaT cell lines (IC_{50} = 33 ± 8 µg/mL on B16 and IC_{50} = 23 ± 5 µg/mL on HaCaT) and a decrease of the activity of RE against B16 (IC_{50} > 200 µg/mL). This experiment confirms nevertheless that the culture conditions used are optimal for the cytotoxicity of the extracts from bioreactor.

Experimental design in Erlenmeyer flasks

The data obtained in Erlenmeyer flasks were analyzed in a same manner using a Taguchi array and the yields and cytotoxic activities of the both extracts (RE and SE) were measured on B16 and HaCaT cell lines (Table 10).

Table 10. Extraction yield and cytotoxicity of the extracts on B16 and HaCaT cell lines for the experimental design in Erlenmeyer flasks.

Exp n°	RE			SE		
	Yield (mg/L)	IC_{50} (µg/mL)		Yield (mg/L)	IC_{50} (µg/mL)	
		B16	HaCaT		B16	HaCaT
**E1**	306.7	280 ± 160	110 ± 60	199.3	375 ± 110	40 ± 15
**E2**	279.7	96 ± 9	122 ± 4	77.9	73 ± 20	77 ± 16
**E3**	218.7	70 ± 8	71 ± 19	66.0	74 ± 34	56 ± 24
**E4**	96.3	145 ± 15	122 ± 22	74.0	380 ± 105	75 ± 50
**E5**	184.0	130 ± 4	127 ± 13	57.3	390 ± 100	240 ± 150
**E6**	305.7	64 ± 8	72 ± 1	58.3	170 ± 10	152 ± 18
**E7**	202.0	**6 ± 0.5**	30 ± 7	583.3	480 ± 230	140 ± 70
**E8**	176.7	81 ± 10	50 ± 7	840.3	>500	260 ± 110
**E9**	489.0	63 ± 7	79 ± 15	39.7	137 ± 7	32 ± 10

The most important cytotoxic activity was demonstrated with experiment **E7** (RE, IC_{50} = 6 ± 0.5 µg/mL on B16) with the following culture conditions: pH 6, temperature 37°C and 5% inoculum and led to a sufficient amount of extract (202.0 mg/L) (Table 10). Interestingly, these conditions were close to those obtained for the culture in bioreactor (excepted for the

value of stirring fixed at 150 rpm for Erlenmeyer process), confirming that these parameters are the best to improve the cytotoxicity of the extracts. Conversely, the highest extraction yield (840.3 mg/mL) was obtained with **E8** and the conditions (37°C, pH 7 and inoculum 1%, table 10) were completely different than those obtained for culture in bioreactor.

Nevertheless, the ANOVA analysis of the results obtained from these Erlenmeyer cultures showed that none of the selected parameters had a significant effect on the cytotoxicity of the extracts on B16 cell lines or on the extraction yield.

Impact of the culture vessel on the activity of extracts

In order to determine the effect of the culture conditions on the production of cytotoxic metabolites, we compared the results obtained for three processes performed using either bioreactor or Erlenmeyer flasks and possessing similar culture conditions with a stirring speed fixed at 150 rpm (Table 11). Among them, the most active extracts were obtained at 37 °C, pH 6 and a 2% inoculum ratio (experiments **B9** and **E9**). The yields were also higher in these experiments in comparison to the other ones.

Table 11. Comparison of the results obtained for cultures in bioreactor and in Erlenmeyer flasks with the same culture conditions.

Experiences	Factors			RE			SE		
	Temperature (°C)	pH	Inoculum ratio (%)	Yield (mg/L)	IC_{50} (µg/mL)		Yield (mg/L)	IC_{50} (µg/mL)	
					B16	HaCaT		B16	HaCaT
**B1**	25	6	1	146.1	157 ± 77	92 ± 29	46.3	>500	460 ± 140
**E1**	25	6	1	306.7	280 ± 160	110 ± 60	199.3	375 ± 110	40 ± 15
**B5**	30	7	5	177.8	345 ± 95	120 ± 40	506.9	100 ± 30	39 ± 10
**E5**	30	7	5	184.0	130 ± 4	127 ± 13	57.3	390 ± 100	240 ± 150
**B9**	37	8	2	**318.0**	**90 ± 50**	**110 ± 25**	**43.9**	**390 ± 90**	**45 ± 2**
**E9**	37	8	2	**489.0**	**63 ± 7**	**79 ± 15**	**39.7**	**137 ± 7**	**32 ± 10**

Comparison of HPLC profiles of the most active extracts

The HPLC chromatograms of these two experiments **B9** and **E9** were compared in Fig 2. Those of the most active extracts obtained during this study (SE of the large scale culture in TY shown in preliminary assays, SE from **B3** and RE from **E7**) were reported in Fig 3. The compounds previously isolated from this *Nocardia* strain [16] and known compounds such as cyclo (L-Ala-L-Phe) [21], adenine, adenosine and 1-(5-deoxy-β-D-erythro-pent-4-enofuranosyl) [22] were identified by comparison with a standard and data from literature (Figs 2A, 2B and 3).

The main differences between the production of metabolites by the strain in the RE of **B9** and **E9** are due to the presence of an unidentified compound with a r.t. of 19 min and of a diketopiperazine cyclo-(L-Ala-L-Phe) which were produced in large amount in bioreactor **B9** but not in Erlenmeyer flask **E9** (Fig 2A). Variability was also observed for the production of compounds eluted between 26 and 32 min (Fig 2A). In the SE of **E9** a compound eluted at the beginning of the gradient appeared. Moreover, a difference of the production of compounds eluted between 22 and 28 min was also highlighted between the two culture vessels (Fig 2B). The production of adenine was most important in bioreactor than in Erlenmeyer reaction set-up (Fig 2). Despite these differences in the chemical profiles, the activity of the extracts were similar between bioreactor and Erlenmeyer flasks (except for the SE on B16 cells) meaning that the compounds highlighted above are not implicated in the cytotoxicity of the extracts.

Fig 2. Comparison of chemical profiles of B9 (red) and E9 (blue). A. Resin extracts, B. Supernatant extracts. All samples were analysed at 220 nm on Prevail® reversed phase C18 column with a gradient of H_2O (A)/acetonitrile (B) (10 min 100% of A, 30 min from 0% of B to 100% of B, 10 min 100% of B)

Finally, the comparison of the most active extracts led to the highlighting of unidentified compounds common in all the three active extracts which were indicated with black squares in Fig 3.

Fig 3. Comparison of the chemical profiles of the most active extracts. SE of the large scale culture in TY shown in preliminary assays (red), SE of **B3** (blue) and RE of **E7** (black). All samples were analysed at 220 nm on Prevail® reversed phase C18 column with a gradient of H_2O (A)/acetonitrile (B) (10 min 100% of A, 30 min from 0% of B to 100% of B, 10 min 100% of B).

Discussion

Lichens are a novel ecological niche of considerable interest for the discovery of bacterial strains with biotechnological potential [12]. We selected and studied a lichen-associated Actinobacterium, *Nocardia* sp that demonstrated interesting cytotoxic activities. The results detailed herein showed that the production of cytotoxic compounds by this *Nocardia* sp. could be modulated by culture conditions according to the OSMAC (One Strain/Many Compounds) approach previously described [23]. The data obtained in preliminary assays are in accordance with our expectations. The marine broth currently used in our experiments performed on strains isolated from marine lichens [12,24] is not suitable to optimize the cytotoxicity of our *Nocardia* strain isolated from a terrestrial lichen. Moreover, the amount of production of metabolites increases with rich medium such as TY. This observation is close to those of Abdelmohsen et al [25] who indicated that the production

of actinosporin by a marine sponge associated-Actinokineospora was widely affected depending on the culture medium used. Our results suggest in the same manner that the production of the compound(s) involved in the cytotoxicity of the extracts is influenced by the difference in the medium growth used.

A significant effect of temperature on cytotoxicity of the resin extracts ($p < 0.05$) with an improvement of the activity at 37°C was exhibited by ANOVA analysis of the data from experimental design in bioreactor. The importance of culture parameters such as temperature on increasing of the biomass and of the activity has been already demonstrated for Actinobacteria strains. Thus, the effect of the temperature has been described on the biomass and geosmin production by strains of *N. cummidelens* and *N. fluminea*. The highest biomass production was thus obtained at the warmest temperature tested, 30°C and 25°C, for the both strains and the production of geosmin was optimal at 25°C [26]. Similar effects has been reported on the production of rapamycin by a *Streptomyces* strain, where the optimal temperature was established at 23°C, the lowest tested value [27].

In spite of employing similar culture conditions (temperature 37°C, pH 6 and inoculum 5%,) in both the bioreactor (**B3**) and Erlenmeyer flasks (**E7**), the best activity (IC_{50} = 6 ± 0.5 µg/mL on B16) was displayed by extracts obtained from Erlenmeyer flasks (**E7**) highlighting the importance of the culture process used.

Various compounds have been previously isolated from the culture broth of this bacterial strain [16]. Compounds identified were mainly diketopiperazines. An auxin derivative and purine derivatives (e.g. adenine) have also been isolated (data not published) but none of these compounds explained the cytotoxic activity of the bacterial extracts.

The comparison of the chemical profiles of extracts obtained by culture in bioreactor and in Erlenmeyer flasks highlighted several differences which also revealed the impact of the system used. These difference of production could be explained by the different aeration

and the stirring system used [28]. In fact, the stirring in bioreactor is performed by agitation blade submerged in the culture broth which implicates hyphal breaks, while in shaken flasks the agitation is performed with an orbital shaker allowing the hyphal formation. In a similar way, the aeration system in bioreactor consisted of a tube submerged in the culture broth that conducted oxygen into the medium causing the formation of bubbles, while in the shaken flasks there is no specific system for aeration. These main differences could explain the variation of bacterial production profile due to a different oxygenation and stirring methods inducing different stress conditions for bacteria.

The comparison of the chemical profiles of the most active extracts (Fig 3) showed a very similar production and the major compounds already isolated possess no cytotoxic properties on B16 cell line. Nevertheless, some zones of the chromatogram corresponding to unidentified compounds have been highlighted (Fig 3).

Conclusion

In conclusion, the Taguchi method for design of experiences combined with an ANOVA test were shown to be powerful tools for the optimization of culture parameters of lichen-associated *Nocardia* sp. for the production of cytotoxic compounds. Further separative experiments will be carried out with a focus on the zones highlighted on HPLC profiles during this study in order to identify compound(s) involved in the cytotoxic activity of these bacterial extracts.

Acknowledgements

We thank the “Ligue contre le cancer” foundation for the financial support which allowed the acquisition of a bioreactor BioFlo 115® in the laboratory. We sincerely thank L.

Intertaglia (LBBM, Banyuls/mer) for the identification of the strain DP94 and Dr S. Sabbani for the reading and the improvement of English langage.

Abbreviations:

OSMAC, One Strain Many Compounds; **TY**, Tryptone Yeast medium; **LBm**, modified Lysogeny Broth; **MB**, Marine Broth; **ISP2**, International Streptomyces Project 2; **IC_{50}**, half maximal inhibitory concentration; **ANOVA**, Analysis of Variance; **DO**, Dissolved Oxygen; **DOE**, Design of Experiments; **DMSO**, Dimethylsulfoxyde; **NaCl**, sodium chloride; **MeOH**, methanol; **EtOAc**, ethyl acetate; **RE**, resin extract; **SE**, supernatant extract; **NaOH**, sodium hydroxide; **H_2SO_4**, sulfuric acid; **OD**, Optical density; **RPMI**, Roswell Park Memorial Institute medium; **FCS**, fetal calf serum; **MTT**, 3-[4,5-dimethylthiazol-2-yl]-2,5-diphenyltetrazolium bromide; **HPLC**, High Performance Liquid Chromatography; **vvm**: volume of gas per volume of liquid per minute

References

1. Barka EA, Vatsa P, Sanchez L, Gaveau-Vaillant N, Jacquard C, Klenk H-P, et al. Taxonomy, Physiology, and Natural Products of Actinobacteria. Microbiol Mol Biol Rev. 2016;80: 1–43. doi:10.1128/MMBR.00019-15

2. Burg RW, Miller BM, Baker EE, Birnbaum J, Currie SA, Hartman R, et al. Avermectins, New Family of Potent Anthelmintic Agents: Producing Organism and Fermentation. Antimicrob Agents Chemother. 1979;15: 361–367.

3. Linke HA, Mechlinski W, Schaffner CP. Production of amphotericin B-14C by Streptomyces nodosus fermentation, and preparation of the amphotericin B-14C-methyl-ester. J Antibiot (Tokyo). 1974;27: 155–160.

4. Lacal JC, Vázquez D, Fernandez-Sousa JM, Carrasco L. Antibiotics that specifically block translation in virus-infected cells. J Antibiot (Tokyo). 1980;33: 441–446.

5. Umezawa H, Ishizuka M, Maeda K, Takeuchi T. Studies on bleomycin. Cancer. 1967;20: 891–895. doi:10.1002/1097-0142(1967)20:5<891::AID-CNCR2820200550>3.0.CO;2-V

6. Nemoto A, Hoshino Y, Yazawa K, Ando A, Mikami Y, Komaki H, et al. Asterobactin, a new siderophore group antibiotic from Nocardia asteroides. J Antibiot (Tokyo). 2002;55: 593–597.

7. Bilyk O, Luzhetskyy A. Metabolic engineering of natural product biosynthesis in actinobacteria. Curr Opin Biotechnol. 2016;42: 98–107. doi:10.1016/j.copbio.2016.03.008

8. Axenov-Gibanov DV, Voytsekhovskaya IV, Tokovenko BT, Protasov ES, Gamaiunov SV, Rebets Y, et al. Actinobacteria Isolated from an Underground Lake and Moonmilk Speleothem from the Biggest Conglomeratic Karstic Cave in Siberia as Sources of Novel Biologically Active Compounds. PLOS ONE. 2016;11. doi:10.1371/journal.pone.0149216

9. Aschenbrenner IA, Cernava T, Berg G, Grube M. Understanding Microbial Multi-Species Symbioses. Front Microbiol. 2016;7. doi:10.3389/fmicb.2016.00180

10. Suzuki MT, Parrot D, Berg G, Grube M, Tomasi S. Lichens as natural sources of biotechnologically relevant bacteria. Appl Microbiol Biotechnol. 2016;100: 583–595. doi:10.1007/s00253-015-7114-z

11. Selbmann L, Zucconi L, Ruisi S, Grube M, Cardinale M, Onofri S. Culturable bacteria associated with Antarctic lichens: affiliation and psychrotolerance. Polar Biol. 2010;33: 71–83. doi:10.1007/s00300-009-0686-2

12. Parrot D, Antony-Babu S, Intertaglia L, Grube M, Tomasi S, Suzuki MT. Littoral lichens as a novel source of potentially bioactive Actinobacteria. Sci Rep. 2015;5: 15839. doi:10.1038/srep15839

13. Bates ST, Cropsey GWG, Caporaso JG, Knight R, Fierer N. Bacterial Communities Associated with the Lichen Symbiosis. Appl Environ Microbiol. 2011;77: 1309–1314. doi:10.1128/AEM.02257-10

14. Grube M, Cardinale M, de Castro JV, Müller H, Berg G. Species-specific structural and functional diversity of bacterial communities in lichen symbioses. ISME J. 2009;3: 1105–1115. doi:10.1038/ismej.2009.63

15. Parrot D, Legrave N, Delmail D, Grube M, Suzuki M, Tomasi S. Review – Lichen-Associated Bacteria as a Hot Spot of Chemodiversity: Focus on Uncialamycin, a Promising Compound for Future Medicinal Applications. Planta Med. 2016;82: 1143–1152. doi:10.1055/s-0042-105571

16. Noël A, Ferron S, Rouaud I, Gouault N, Hurvois J-P, Tomasi S. Isolation and Structure Identification of Novel Brominated Diketopiperazines from Nocardia ignorata—A Lichen-Associated Actinobacterium. Molecules. 2017;22: 371. doi:10.3390/molecules22030371

17. Bhattacharya A, Das S, Majumder P, Batish A. Estimating the effect of cutting parameters on surface finish and power consumption during high speed machining of AISI 1045 steel using Taguchi design and ANOVA. Prod Eng. 2009;3: 31–40.

18. Kim O-S, Cho Y-J, Lee K, Yoon S-H, Kim M, Na H, et al. Introducing EzTaxon-e: a prokaryotic 16S rRNA gene sequence database with phylotypes that represent uncultured species. Int J Syst Evol Microbiol. 2012;62: 716–721. doi:10.1099/ijs.0.038075-0

19. Shirling EB, Gottlieb D. Methods for characterization of Streptomyces species1. Int J Syst Evol Microbiol. 1966;16: 313–340. doi:10.1099/00207713-16-3-313

20. Bézivin C, Tomasi S, Lohézic-Le Dévéhat F, Boustie J. Cytotoxic activity of some lichen extracts on murine and human cancer cell lines. Phytomedicine. 2003;10: 499–503. doi:10.1078/094471103322331458

21. Cho JY, Kang JY, Hong YK, Baek HH, Shin HW, Kim MS. Isolation and Structural Determination of the Antifouling Diketopiperazines from Marine-Derived Streptomyces praecox 291-11. Biosci Biotechnol Biochem. 2012;76: 1116–1121. doi:10.1271/bbb.110943

22. Perrone P, Daverio F, Valente R, Rajyaguru S, Martin JA, Lévêque V, et al. First Example of Phosphoramidate Approach Applied to a 4‘-Substituted Purine Nucleoside (4‘-Azidoadenosine): Conversion of an Inactive Nucleoside to a Submicromolar Compound versus Hepatitis C Virus. J Med Chem. 2007;50: 5463–5470. doi:10.1021/jm070362i

23. Bode HB, Bethe B, Höfs R, Zeeck A. Big Effects from Small Changes: Possible Ways to Explore Nature’s Chemical Diversity. ChemBioChem. 2002;3: 619–627. doi:10.1002/1439-7633(20020703)3:7<619::AID-CBIC619>3.0.CO;2-9

24. Parrot D, Intertaglia L, Jehan P, Grube M, Suzuki MT, Tomasi S. Chemical analysis of the Alphaproteobacterium strain MOLA1416 associated with the marine lichen Lichina pygmaea. Phytochemistry. 2018;145: 57–67. doi:10.1016/j.phytochem.2017.10.005

25. Abdelmohsen UR, Cheng C, Viegelmann C, Zhang T, Grkovic T, Ahmed S, et al. Dereplication Strategies for Targeted Isolation of New Antitrypanosomal Actinosporins A and B from a Marine Sponge Associated-Actinokineospora sp. EG49. Mar Drugs. 2014;12: 1220–1244. doi:10.3390/md12031220

26. Schrader KK, Harries MD, Page PN. Temperature effects on biomass, geosmin, and 2-methylisoborneol production and cellular activity by Nocardia spp. and Streptomyces spp. isolated from rainbow trout recirculating aquaculture systems. J Ind Microbiol Biotechnol. 2015;42: 759–767. doi:10.1007/s10295-015-1600-2

27. Dutta S, Basak B, Bhunia B, Sinha A, Dey A. Approaches towards the enhanced production of Rapamycin by Streptomyces hygroscopicus MTCC 4003 through mutagenesis and optimization of process parameters by Taguchi orthogonal array methodology. World J Microbiol Biotechnol. 2017;33: 90. doi:10.1007/s11274-017-2260-3

28. Jia B, Jin ZH, Mei LH. Medium Optimization Based on Statistical Methodologies for Pristinamycins Production by Streptomyces pristinaespiralis. Appl Biochem Biotechnol. 2008;144: 133. doi:10.1007/s12010-007-8012-3

Supporting information

S1 Fig: Monitoring of bacterial growth of scale-up in TY and LBm.

S2 Fig: Monitoring of culture parameters of B1.

S3 Fig: Monitoring of bacterial growth of B1.

S4 Fig: Monitoring of bacterial growth of E1.

 Click here to download Figure Fig1.tif

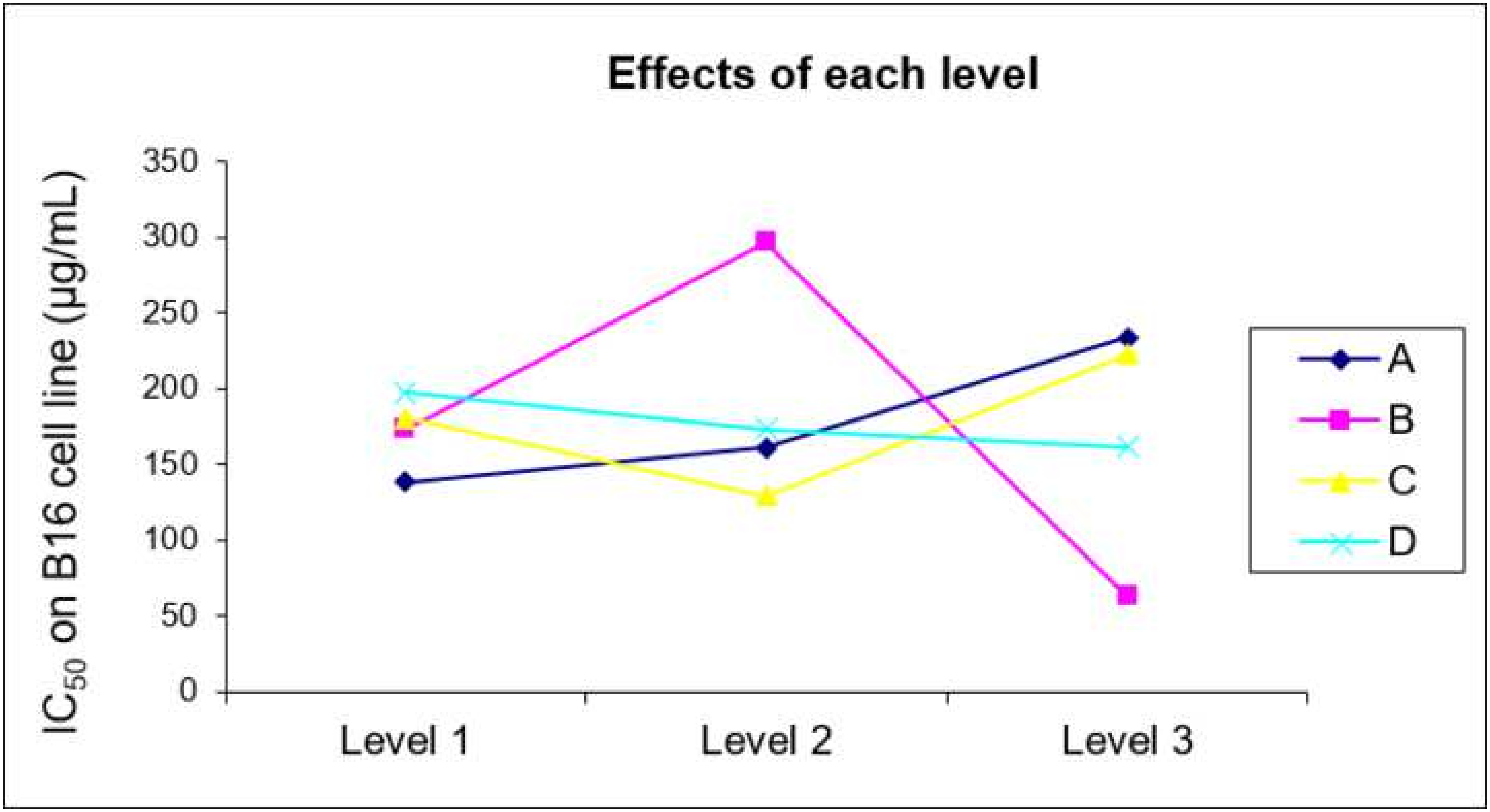

Figure 2

Click here to download Figure Fig2.tif

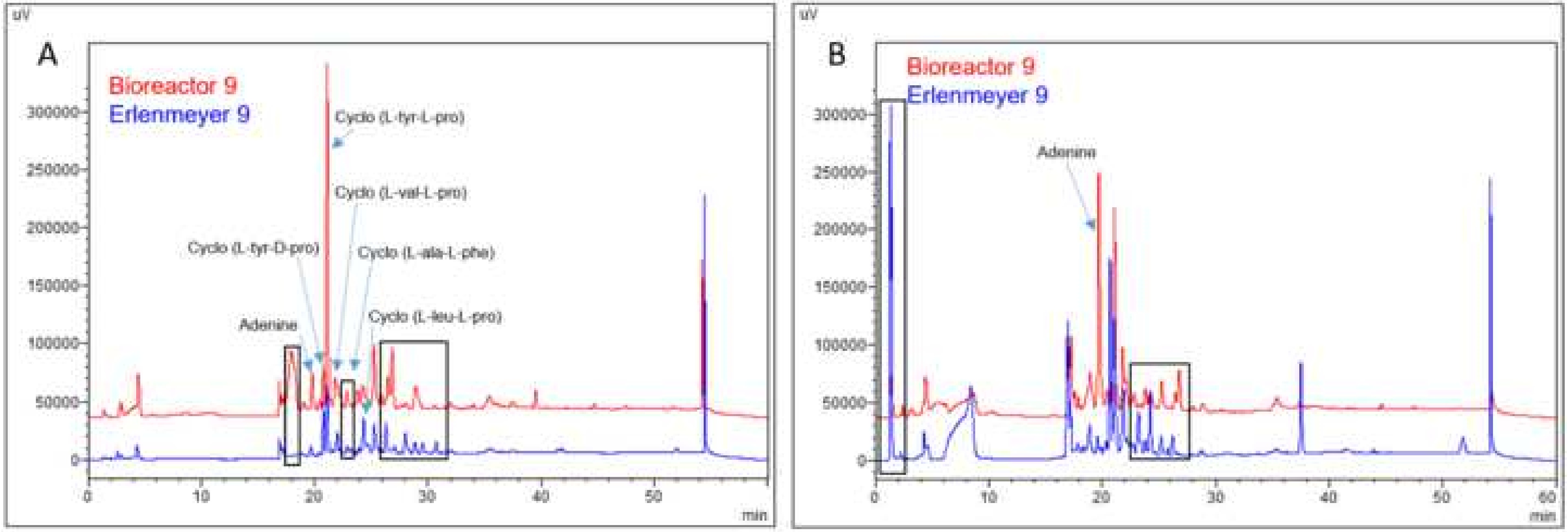

Figure 3

Click here to download Figure Fig3.tif

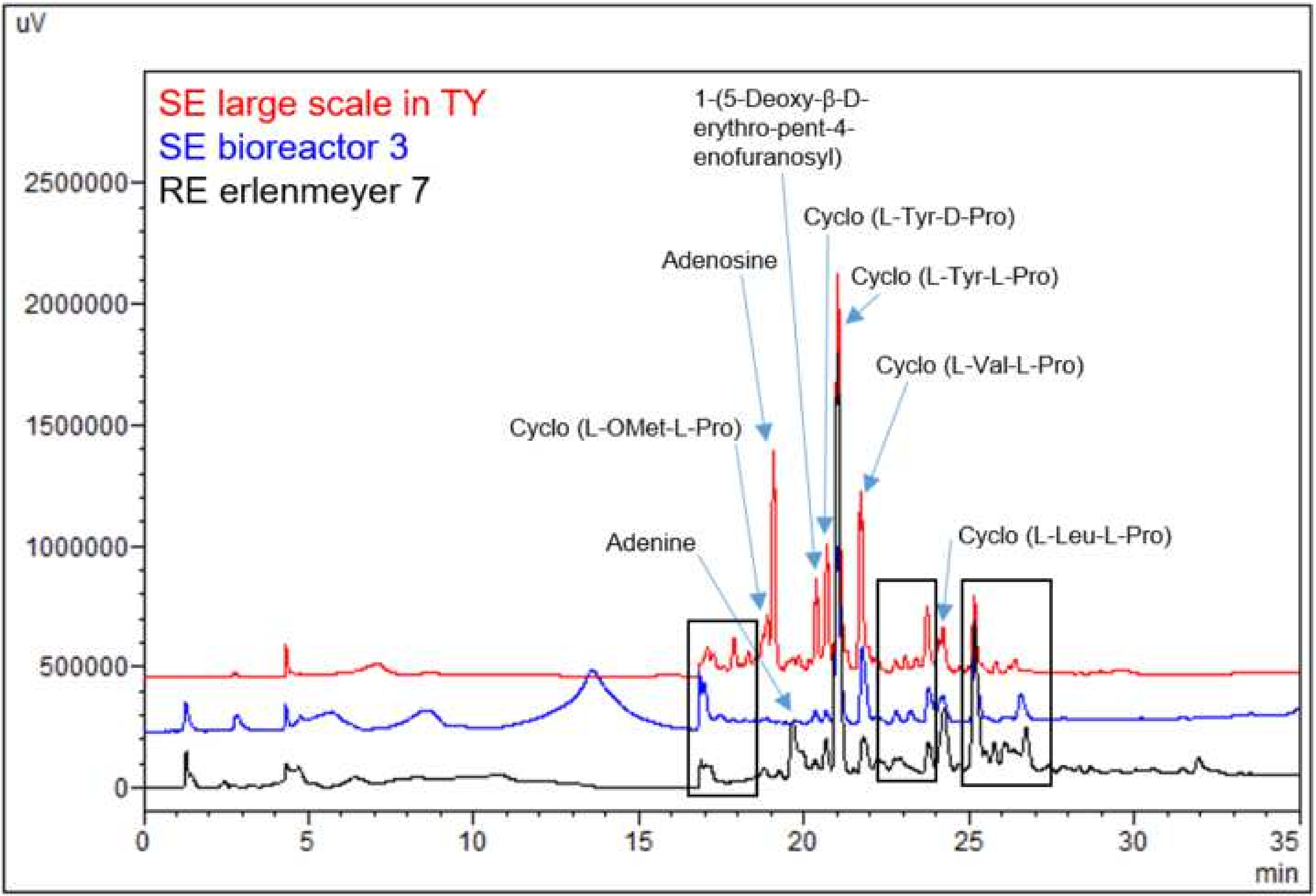

S1 Fig

Click here to access/download
Supporting Information
S1_Fig.tif

S2 Fig

Click here to access/download
Supporting Information
S2_Fig.tif

S3 Fig

Click here to access/download
Supporting Information
S3_Fig.tif

S4 Fig

Click here to access/download
Supporting Information
S4_Fig.tif
